# Radiotherapy and inflammaging: the influence of prostate cancer radiotherapy on systemic inflammation

**DOI:** 10.1007/s00345-024-05409-z

**Published:** 2024-12-18

**Authors:** Katarzyna Paal, Bettina Stranz, Eva-Maria Thurner, Tobias Niedrist, Wilfried Renner, Tanja Langsenlehner

**Affiliations:** 1 Department of Therapeutic Radiology and Oncology, Comprehensive Cancer Center, Medical University of Graz, 8036 Graz, Austria; 2 Clinical Institute of Medical and Chemical Laboratory Diagnostics, Medical University of Graz, 8036 Graz, Austria

**Keywords:** Prostate cancer, Radiation therapy, Inflammation, aging, Inflammaging, Malnutrition

## Abstract

**Purpose:**

The present study was performed to investigate the association of prostate cancer radiotherapy with inflammaging, a condition characterized by the elevation of inflammatory blood parameters that significantly increases the susceptibility to the occurrence or progression of age-related conditions.

**Patients and Methods:**

A total of 306 patients treated with curative radiotherapy (RT) for prostate cancer were enrolled into the prospective study. Aging-related inflammatory parameters including C-reactive protein (CRP), albumin, fibrinogen, cholesterol, platelet-to-lymphocyte ratio (PLR), and neutrophil-to-lymphocyte ratio (NLR) were analyzed before and at the end of RT, and 3 and 15 months after completion of the RT. Statistical analysis was performed using non-parametric variance analysis.

**Results:**

Overall variance analysis showed a significant influence of RT on all inflammatory parameters (p < 0.001) with the exception of CRP (p = 0.498). Pairwise analysis revealed a significant elevation of fibrinogen (p = 0.041), NLR (p < 0.001), and PLR levels (p < 0.001) as well as a significant decrease of albumin (p < 0.001) and cholesterol levels (p < 0.001) during the RT course. After completion of RT, a significant recovery was detected for NLR, PLR, albumin and cholesterol. However, 15 months after RT, PLR, fibrinogen, and cholesterol remained significantly lower when compared to the baseline (p < 0.001).

**Conclusion:**

Our results indicate that radiation therapy triggers chronic inflammatory processes that could contribute to the development, acceleration or worsening of age-related alterations and conditions. Further investigations to estimate the long-term consequences of curative radiation therapy on clinical manifestations of aging are warranted.

**Supplementary Information:**

The online version contains supplementary material available at 10.1007/s00345-024-05409-z.

## Introduction

Radiotherapy is a highly effective prostate cancer treatment that utilizes high-energy radiation to eliminate cancer cells. It is a well-established and efficient non-surgical option even for prostate cancer patients who are not candidates for radical prostatectomy due to advanced age, comorbidities, or decreased performance status. The number of patients, especially those aged ≥ 70 years, referred for radiotherapy has increased significantly in recent years. While effective in killing cancer cells, RT can also damage surrounding healthy tissues leading to an inflammatory response. Over time, chronic inflammation can cause cellular damage, impair tissue function, and contribute to the development or progression of various age-related conditions.

“Inflammaging” represents a condition characterized by elevated inflammatory blood parameters known to be a key driver of the occurrence of various age-related conditions and the aging process itself. Several reports have described an increase in pro-inflammatory cytokines with increasing age even in healthy elderly persons without apparent presence of acute inflammation [1–3]. Pro-thrombotic factors have also been noted to be increased with chronic inflammation, likely because of a co-stimulatory effect between the two processes [3]. Additionally, low serum cholesterol and albumin levels have been suggested to indicate subclinical levels of chronic inflammation and/or dysregulation of the inflammatory processes that can contribute to an “aging” phenotype [4]. Furthermore, it has been demonstrated that the degree of clinical frailty independently correlates with increased levels of inflammatory markers that have also been associated with mortality in the elderly [5–7].

Cancer treatments may lead to the premature onset of age-related chronic conditions. However, little is known about aging-associated consequences of cancer treatment and the processes that underlie differential responses to therapy, particularly in older adults with comorbidities and higher levels of frailty. Evidence from preclinical models shows that radiation and cytotoxic anticancer therapies activate chronic inflammation and cellular processes that drive accelerated aging [8].

Some theories propose that radiotherapy-induced damage to healthy tissues may lead to a persistent inflammatory response over time. Thus, radiotherapy may represent a potential trigger of inflammaging and contribute to the development or acceleration of age-related alterations and conditions. However, direct evidence is limited. Therefore, this study aimed to examine the effects of radiotherapy on inflammatory processes in older adults undergoing radiation therapy for prostate cancer.

## Patients and methods

This prospective observational study was performed including 306 prostate cancer patients treated at the Department of Therapeutic Radiology and Oncology, Comprehensive Cancer Center, Medical University of Graz. Patients who were 65 years of age or older, candidates for definitive radiation treatment, and gave written informed consent were considered eligible for inclusion. Upon enrollment in the cohort, all participants completed a standard clinical questionnaire, covering aspects such as family medical history, current medications, past surgeries, comorbidities, and smoking habits.

Image-guided radiotherapy with high-energy photons generally involved the utilization of volumetric intensity modulated arc therapy (VMAT) techniques to cover the prostate and seminal vesicles, as deemed appropriate based on clinical considerations. The total dose, prescribed to the International Commission on Radiation Units and Measurement point, ranged from 70 to 78 Gy, administered in 2 Gy per fraction, depending on the risk situation. Thirteen patients with low- or intermediate-risk prostate cancer received hypofractionated radiotherapy, with a total dose ranging from 57 to 60 Gy (3 Gy per fraction). Treatment was performed daily, five days per week.

The study complied with the Declaration of Helsinki and was performed according to the national law. The protocol has been approved by the local Ethical Committee (approval number: EK 31–437 ex 18/19). Written informed consent was obtained from all participants.

Laboratory parameters were assessed at baseline (within 2 weeks prior to initiation of radiation therapy), at the end of radiotherapy as well as 3 and 15 months after completion of radiotherapy. The blood parameters analyzed in the present study included fibrinogen, cholesterol, albumin, and c-reactive protein (CRP). Additionally, the blood count was assessed to estimate the platelet-to-lymphocyte ratio (PLR), calculated as the quotient of absolute platelet count to absolute lymphocyte count, and the neutrophil-to-lymphocyte ratio (NLR), calculated as the quotient of absolute neutrophil count to absolute lymphocyte count.

The normal ranges for the albumin, fibrinogen, and CRP levels were 3.5–5.3 g/dL, 210–400 mg/dL, and −5.0 mg/L, respectively. For cholesterol, the reference value was −200 mg/dL, however, previously, “hypocholesterolemia”, defined as serum cholesterol levels less than 160 mg/dL, has been associated with malnutrition risk. For the NLR and PLR, various reference values have been proposed in the literature. Wang et al. reported reference values for the PLR as 0–170.75 in healthy adults aged 60–69 years and 0–169.91 in those aged 70–79 years. For the NLR, the reference values were 0–2.99 for individuals aged 60–69 years and 0–3.17 for those aged 70–79 years, respectively [9].

### Statistical analysis

The primary endpoint was defined as the development of alterations in inflammatory laboratory parameters during the observational time. The results of inflammatory parameters were demonstrated with the use of descriptive statistics. The influence of radiotherapy on inflammatory markers was analyzed using non-parametric tests for paired samples. P-values ≤ 0.05 were considered statistically significant. Statistical analyses were carried out using SPSS 28.0 for Windows (SPSS inc. 1989).

## Results

### Study cohort

The present analysis included 306 patients treated with definitive radiotherapy for prostate cancer. Median age at start of radiotherapy was 73 years (mean 73.55 ± 5.58). Patient and treatment characteristics are presented in Table 1.

**Table 1:** Patient and treatment characteristics

Parameter	N (%)
Age	
≤ 74	162(52.9%)
75–84	140(45.8%)
≥ 85	4(1.3%)
Smoking status	
Current	13 (4.1%)
Not current	300 (95.2%)
Hypertension	
Yes	177 (56.2%)
No	136 (43.2%)
Diabetes	
Yes	56 (17.8%)
No	257 (81.6%)
Comorbidities	
CCI 0	187 (59.4%)
CCI ≥ 1	97 (30.8%)
Risk group	
High risk	140 (45.8%)
Intermediate risk	138 (45.1%)
Low risk	28 (9.2%)
Tumour stage	
T1-T2	251 (82%)
T3-T4	51 (16.7%)
Missing data	4 (1.3%)
Gleason Score	
≤ 6	48 (15.7%)
7	147 (48%)
8–10	111 (36.3%)
Initial PSA level [ng/ml]	
< 10	202(66%)
10–20	71(23.2%)
> 20	29(9.5%)
Median (mean ± SD)	8.2 (13.4 ± 24.5)
Missing data	4(1.3%)
Use of neoadjuvant ADT	
Yes	286 (93.5%)
No	18 (5.9%)
Missing data	2 (0.7%)
Daily RT dose [Gy]	
1.8–2.0	293 (95.8%)
> 2.0	13 (4.2%)
Total RT dose [Gy]	
≤ 74	35 (11.4%)
76	86 (28.1%)
≥ 78	185 (60.5%)
Median (mean ± SD)	78 Gy (76.2 ± 4)

*ADT* androgen deprivation therapy, *Gy* Gray, *PSA* Prostate specific antigen, *RT* radiation therapy, *CCI* Charlson comorbidity index

Follow-up investigations were performed 3 and 15 months after completion of radiotherapy. A total of 261 patients underwent the first follow-up investigation, the second follow-up investigation was performed in 244 patients. Parameters of chronic inflammation were evaluated before, at the end of RT and at both follow-up investigations. The results of inflammatory parameters are displayed in Table 2.

**Table 2:** Results of inflammatory parameters before and at the end of radiotherapy as well as at the follow-up investigations

Parameter	Baseline Median (mean ± SD)	End or RT Median (mean ± SD)	3 months after RT Median (mean ± SD)	15 months after RT Median (mean ± SD)
NLR	2.71 3.05 ± 1.41	3.63 4.04 ± 1.96	3.15 3.51 ± 1.74	2.78 3.15 ± 1.43
PLR	137.03 149.17 ± 63.81	200 214.18 ± 91.14	180 190.78 ± 81.37	159.64 169.81 ± 70.01
CRP [mg/l]	1.5 3.36 ± 9.95	1.4 2.99 ± 4.81	1.7 2.78 ± 4.12	1.5 2.76 ± 4.28
Fibrinogen [mg/dl]	306 315.67 ± 62.99	315.5 318.54 ± 58.59	320 322.97 ± 55.95	316.5 323,46 ± 56,76
Albumin [g/dl]	4.5 4.45 ± 0.25	4.3 4.32 ± 0.23	4.4 4.37 ± 0.25	4.4 4.41 ± 0.28
Cholesterol [mg/dl]	192 192.63 ± 44.35	184 182.89 ± 42.45	189 190.81 ± 44.71	181 183.45 ± 45.55

*NLR* neutrophil to lymphocyte ratio, *PLR* platelet to lymphocyte ratio, *RT* radiation therapy, *SD* standard deviation

### Association between clinical conditions and inflammatory parameters

We detected a significant relationship between the presence of comorbidities and NLR (p = 0.006) and cholesterol levels (p < 0.001) at baseline. However, a significant influence of comorbidites on the changes of NLR and cholesterol levels between the different assessments was not found.

Furthermore, we observed a significant association between diabetes mellitus and cholesterol levels (p < 0.001) as well as an association of hypertension with fibrinogen (p = 0.017) and cholesterol levels (p = 0.014) at baseline. However, a significant influence of diabetes mellitus and hypertension on alterations of fibrinogen or cholesterol levels between the different examinations was not detected. Smoking status was not significantly associated with inflammatory parameters.

### Influence of radiotherapy on chronic inflammation

During the observation period, variance analysis revealed significant differences in the outcome of the NLR (p < 0.001) and PLR levels (p < 0.001). Similarly, significant alterations were detected in the albumin (p < 0.001) as well as fibrinogen levels (p > 0.001). Furthermore, variance analysis demonstrated significant differences in the results of cholesterol levels (p < 0.001) whereas significant changes in the results of CRP levels were not detected (p = 0.498). Results of variance analyses are displayed in Supplementary Information Fig. 1a–f.

In pairwise analyses, a significant increase of the NLR was detected at the end of RT when compared to the baseline (p < 0.001) that was followed by a significant reduction in the NLR levels during follow up (p < 0.001). At the second follow-up examination, no significant difference was observed in comparison to the baseline (p = 0.152).

Furthermore, a significant increase of the PLR was found at the end of RT course in comparison to the baseline (p < 0.001). During follow-up, a significant decrease of PLR levels was noted (p < 0.001). However, at the second follow- up investigation, the PLR was still significantly increased when compared to the baseline (p < 0.001).

In addition, a significant decline of albumin levels at the end of RT course as well as at the first follow-up examination in comparison to the baseline was detected (p < 0.001 for both comparisons, Table 3). A significant recovery in albumin levels at the first and second follow-up when compared to the end of the RT course (p < 0.001 for both comparisons) as well as an increase in albumin between the first and second follow-up were also found (p = 0.032). However, a significant difference between the baseline and the second follow-up was not detected (p = 0.222).

**Table 3:** Results of pairwise comparisons of the analysed parameters

	NLR		PLR		CRP		Fibrinogen		Albumin		Cholesterol	
	Test Statistics*	P***	Test Statistics	P	Test Statistics	P	Test Statistics	P	Test Statistics	P	Test Statistics	P
Baseline –end of RT	– 1.25	** < 0.001**	– 1.762	** < 0.001**	n.s	n.s	– 0.293	**0.041**	0.906	** < 0.001**	0.759	** < 0.001**
	**↑**		**↑**		** – **		**↑**		**↓**		**↓**	
End of RT-3 months after RT	0.613	** < 0.001**	0.652	** < 0.001**	n.s	n.s	– 0.281	**0.05**	– 0.457	** < 0.001**	−0.519	** < 0.001**
	**↓**		**↓**		** – **		**↑**		**↑**		**↑**	
3 months after RT –15 months after RT	0.462	** < 0.001**	0.556	** < 0.001**	n.s	n.s	– 0.127	0.378	– 0.286	**0.032**	0.449	< **0.001**
	**↓**		**↓**		** – **		** – **		**↑**		**↓**	
Baseline –3 months after RT	– 0.637	** < 0.001**	– 1.11	** < 0.001**	n.s	n.s	−0.574	** < 0.001**	0.449	** < 0.001**	0.241	0.071
	**↑**		**↑**		** – **		**↑**		**↓**		** – **	
Baseline –15 months after RT	– 0.176	0.152	– 0.554	** < 0.001**	n.s	n.s	– 0.701	** < 0.001**	0.163	0.222	0.69	< **0.001**
	** – **		**↑**		** – **		**↑**		** – **		**↓**	
P** Overall difference	** < 0.001**		** < 0.001**		0.498		** < 0.001**		** < 0.001**		** < 0.001**	

*NLR* neutrophil to lymphocyte ratio, *PLR* platelet to lymphocyte ratio, *CRP* C-reactive protein, *RT* radiation therapy, *n.s.* not significant

^*^Significant alterations (p < 0.05) as well as the direction of the significant changes (↓↑) were marked in bold

^**^Statistical analyses were performed using related samples Friedman’s two-way analysis of variance by ranks summary

^***^Pairwise comparisons of related-samples Friedman’s two-way analysis of variance

Moreover, a significant increase in fibrinogen levels was found at the end of the RT course (p = 0.041). During follow-up, a further increase of fibrinogen levels was detected, that were significantly elevated at the second follow-up examination when compared to the baseline (p < 0.001).

Pairwise comparisons demonstrated a significant decline in the results of cholesterol level at the end of RT (p < 0.001). Additionally, a significant decline of the cholesterol levels between the first and second follow-up (p < 0.001) could be found. There was also a significant decrease of cholesterol levels at the second follow-up when compared to the baseline (p < 0.001). The results of pairwise comparisons are shown in Table 3.

## Discussion

In the present study, we analyzed the impact of small-volume radiotherapy on inflammatory processes associated with aging in a cohort of older adults treated for prostate cancer and identified significant changes in inflammatory markers. Specifically, we observed significant dynamics in the NLR and PLR levels, along with a significant increase in fibrinogen levels and alterations in albumin and cholesterol levels.

The involvement of inflammation in the aging process and age-related diseases has been established in numerous epidemiological studies. Elevated levels of pro-inflammatory cytokines, chemokines, and other markers of inflammation have been observed as age increases, even among healthy elderly individuals without evident signs of acute inflammation [10, 11].

For instance, elevated PLR and NLR levels have been linked to physiological aging processes and have been identified in older adults [12]. Increased levels of NLR and PLR have also been associated with age- related conditions such as a lower grip strength, and sarcopenia as well as cognitive decline and frailty [13–15].

An elevation in fibrinogen levels has also been noted in relation to aging and age-related conditions. For instance, Welstead et al. identified an association between elevated fibrinogen levels and frailty [16]. Further research has linked elevated fibrinogen levels to an increase in long-term comorbidities [17, 18].

Low serum cholesterol and albumin have been suggested to indicate subclinical levels of chronic inflammation and/or dysregulation of inflammatory processes that can contribute to an “aging” phenotype [3]. A decrease in serum albumin during advanced age has been consistently observed. For instance, among hospitalized elderly individuals, up to 90% of patients were found to have hypoalbuminemia [19] suggesting it is a component of the natural aging process. Lower serum albumin levels have been reported in numerous studies among individuals exhibiting clinical signs of sarcopenia and have been linked to frailty [20].

Low levels of blood cholesterol have also been associated with an increased risk of frailty [21]. Moreover, decreased blood cholesterol levels have been correlated with age-related conditions such as cognitive impairment, poorer somatic health, higher disability, and a higher level of malnutrition [22]. Thus, reduced serum cholesterol levels can be linked to an accelerated aging process, leading to limited compensatory capacity and unfavorable outcomes.

Chronic inflammatory markers have been suggested to predict both functional decline and mortality [23] and aid in identifying patients with functional impairments that may not otherwise have been detected without extensive geriatric assessment testing.

Radiotherapy is a highly effective cancer treatment option. Its effects rely on direct and indirect interactions with cellular DNA that may induce degradation of DNA strands, disrupt DNA synthesis and lead to a significant reduction in cell division potential. As a result, the diminished ability for cell division contributes to a decline in the number of stem and progenitor cells, promoting cellular senescence and triggering chronic inflammation.

Large epidemiologic studies have reported the premature onset of age-related conditions in childhood cancer survivors, decades earlier than in their peers independent of organ-specific, treatment-related toxicities. Adults treated for childhood cancer are at an increased risk of chronic health conditions, frequent hospitalization and early mortality [24–26]. Elevated rates of other outcomes typically associated with aging, such as minimal cognitive dysfunction, reduced muscle strength, and poor exercise tolerance, are also reported to appear decades earlier than expected [27–29].

Although a phenotype of accelerated aging after cancer treatment has been observed, the mechanisms that drive accelerated aging in cancer survivors are not understood. Asymptomatic cancer survivors have been found to demonstrate a biologic profile of chronic inflammation, consistent with an early onset of cellular processes that drive accelerated aging [30, 31].

In our study, we detected significant alterations in parameters linked to chronic inflammation. During radiotherapy, a significant increase in the levels of NLR, PLR, and fibrinogen was observed. Although a noticeable recovery was evident after the completion of the therapy, the PLR and fibrinogen levels remained significantly elevated compared to the baseline situation before the therapy. Additionally, there was a significant decrease in the albumin and cholesterol levels during the therapy. Although the values increased again afterward, the cholesterol level remained significantly lower than baseline. Interestingly, there were no notable changes detected in the levels of CRP.

Our findings support the hypothesis that radiotherapy may contribute to the acceleration of aging related processes. Even though only a small-volume therapy was conducted, significant changes in aging-related inflammatory parameters were found. In previous studies, low albumin and cholesterol levels have been linked with reduced nutritional status [32, 33]. Thus, the alterations in cholesterol and albumin levels might also be associated with an increased risk of developing malnutrition and aging symptoms. However, further research is necessary to confirm or refute these findings.

To the best of our knowledge, our study to date represents the first prospective analysis of the influence of curative small-field radiation therapy on aging-related inflammatory and metabolic processes, measured with biochemical parameters in the peripheral blood. The study cohort included a homogenous patient collective regarding age, cancer type, and prescribed cancer treatment. The analysis of the patients from one tertiary clinical centre contributes to a high homogeneity in the application of radiation therapy, particularly, regarding total radiation dose, target volume delineation, dose constraints for organs at risk, and daily set-up. We also assume, that the prospective study design and observational time of 16–17 months allow for firm conclusions about the long-term influence of RT on the aging process.

The limitation of our study is data collection from only one patients’ collective without a comparison with a non-irradiated, sex- and age matched control group form the same geographical region, which could enable a comparison with the dynamics of natural aging process with the same measures. However, an attempt of constructing the matching control group without any malignancy would result in a strong selection bias and deliver unreliable data about patients’ health. In addition, various biomarkers including interleukin (IL)- 1, IL- 6, transforming growth factor- ß, and tumor necrosis factor- α that have been linked with the inflammatory response, were not considered. However, in our study, we primarily aimed at investigating the influence of radiotherapy on inflammation measured with inflammatory parameters that have previously also been associated with aging- related clinical conditions and that are relatively easy to assess without additional laborious efforts, making them attractive parameters in routine clinical practice.

In conclusion, the data of our study indicate that radiotherapy may induce an inflammatory process that could contribute to the development, acceleration or worsening of age-related alterations and conditions. Nevertheless, further studies are necessary before definitive conclusions on the consequences of curative radiation therapy on the manifestations of aging can be drawn. If additional studies confirm our findings, aging-related effects should be considered in all patients undergoing radiation therapy, particularly those at advanced age or carrying a burden of chronic diseases.

## Supplementary Information

Below is the link to the electronic supplementary material.

Supplementary file1 (DOCX 12 KB)

Supplementary file2 (DOCX 534 KB)

## Data Availability

No datasets were generated or analysed during the current study.
