# Supplementary material for: Radiotherapy and inflammaging: the influence of prostate cancer radiotherapy on systemic inflammation
Source: World J Urol. 2024 Dec 18;43(1):35. doi: 10.1007/s00345-024-05409-z (PMC11655599; doi:10.1007/s00345-024-05409-z)
Supplement: Supplementary file 1 — Supplementary file1 (DOCX 12 KB) [file 345_2024_5409_MOESM1_ESM.docx]

**Supplementary Information**

**Figure caption**

**Figure 1 (a-f).** Related-Samples Friedman's Two-Way Analysis of Variance by Ranks for neutrophil to lymphocyte ratio(a), platelet to lymphocyte ratio(b), albumin(c), fibrinogen (d) and cholesterol (e), and C-reactive protein (f).
