# Supplementary figures and images for: Radiotherapy and inflammaging: the influence of prostate cancer radiotherapy on systemic inflammation
Source: World J Urol. 2024 Dec 18;43(1):35. doi: 10.1007/s00345-024-05409-z (PMC11655599; doi:10.1007/s00345-024-05409-z)

**Supplementary Information**

**Figure 1 (a-f).**

**a
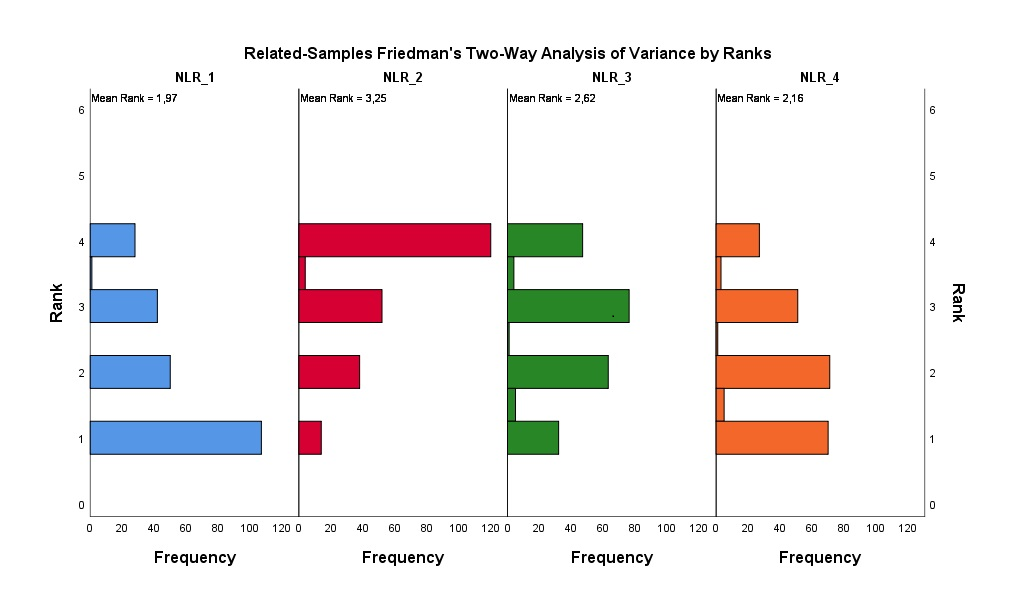
**

**b**

**
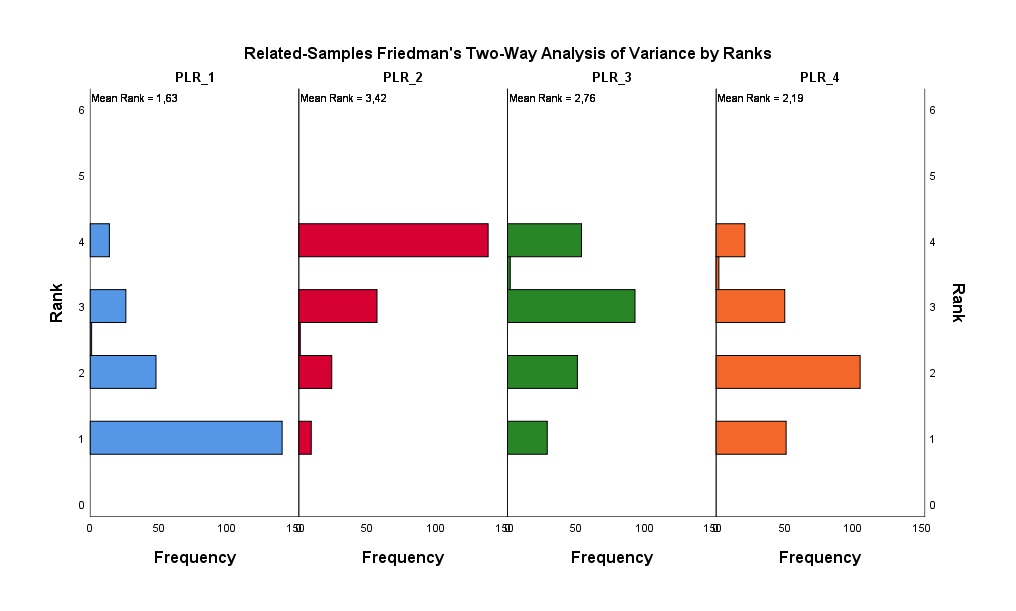
**

**c**

**
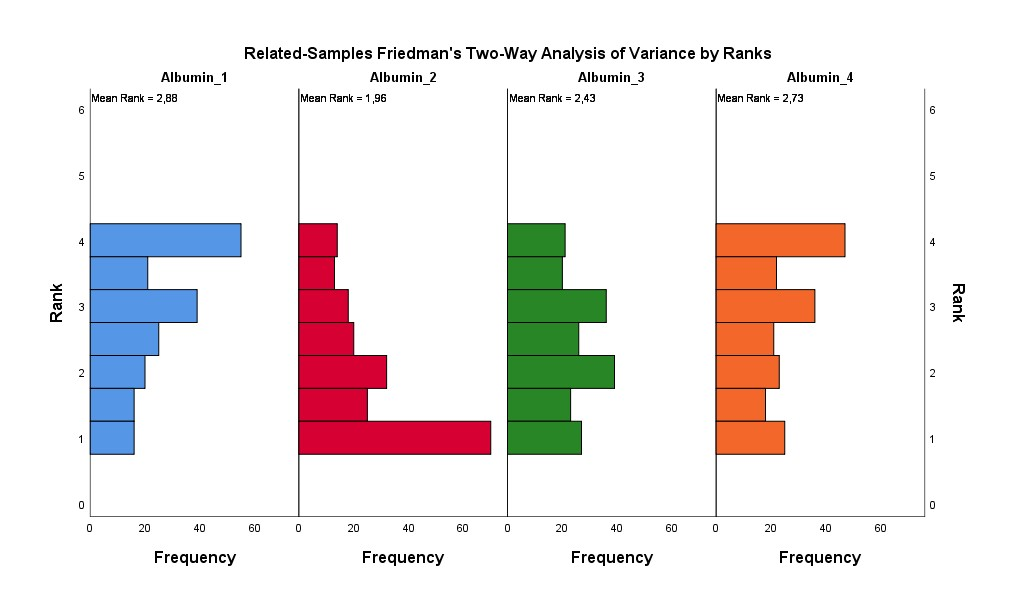
**

**d**

**
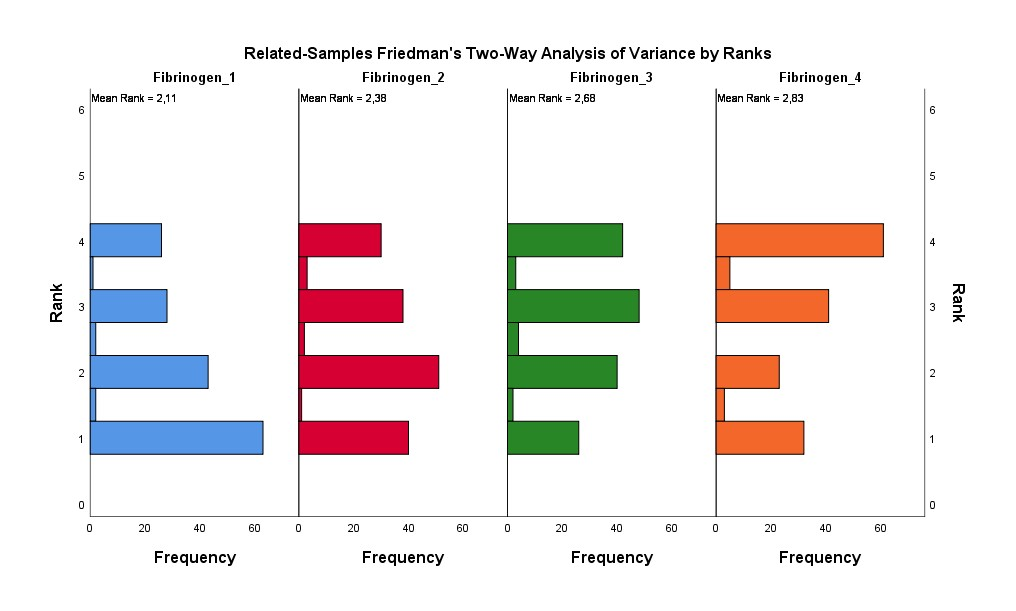
**

**e**

**
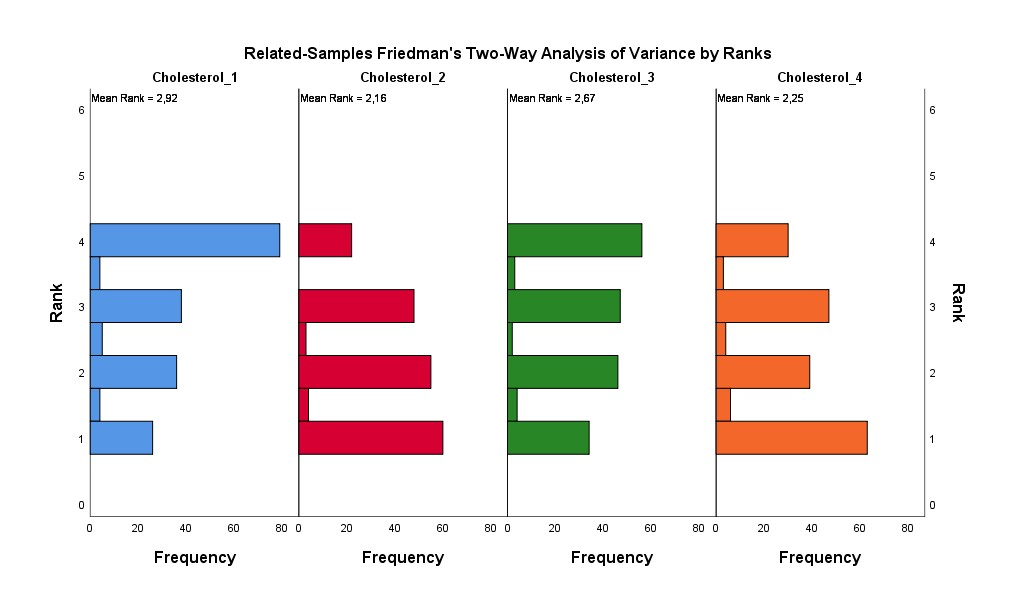
**

**f**

**
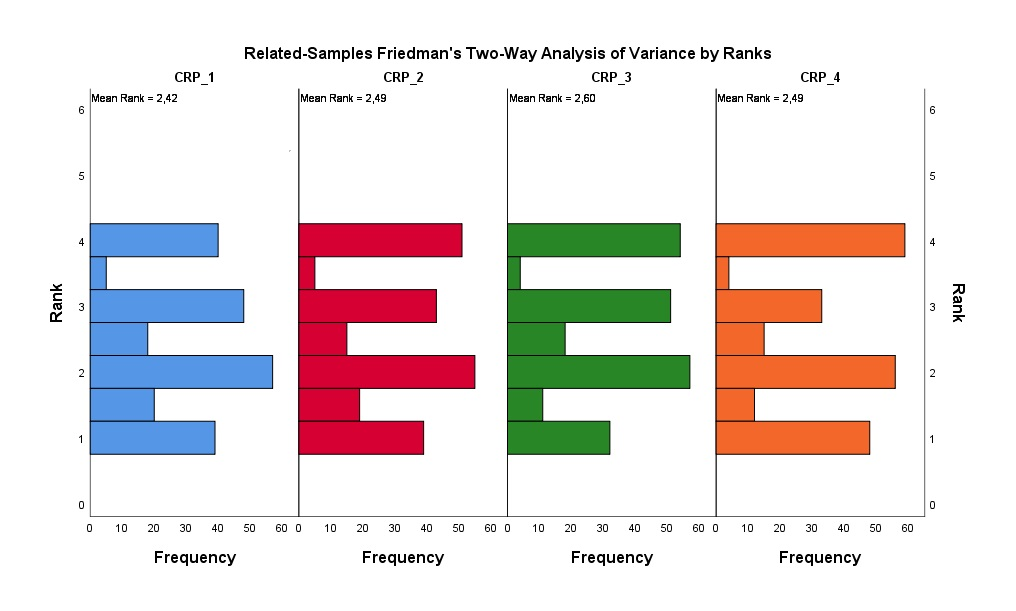
**

Supplement: Supplementary file 2 — Supplementary file2 (DOCX 534 KB) [file 345_2024_5409_MOESM2_ESM.docx]
